# Nutritional Status of Men with Ulcerative Colitis in Remission in a Pair–Matched Case–Control Study

**DOI:** 10.3390/jcm7110438

**Published:** 2018-11-13

**Authors:** Dominika Głąbska, Dominika Guzek, Gustaw Lech

**Affiliations:** 1 Department of Dietetics, Faculty of Human Nutrition and Consumer Sciences, Warsaw University of Life Sciences (SGGW-WULS), 02-776 Warsaw, Poland; 2 Department of Organization and Consumption Economics, Faculty of Human Nutrition and Consumer Sciences, Warsaw University of Life Sciences (SGGW-WULS), 02-776 Warsaw, Poland; dominika_guzek@sggw.pl; 3 Department of General, Gastroenterological and Oncological Surgery, Medical University of Warsaw, 02-097 Warsaw, Poland; gustaw.lech@wum.edu.pl

**Keywords:** *colitis ulcerosa*, body composition, bioelectrical impedance, nutritional status, malnutrition, body mass index

## Abstract

The aim of the presented research was to analyze the nutritional status of male subjects with ulcerative colitis in remission by using body composition that was assessed by bioelectrical impedance measurement against a gender-matched control group. Forty-four male patients in remission with ulcerative colitis were recruited for the case-control study and simultaneously, a matched control group of 44 male individuals without inflammatory bowel diseases was pair-matched (general community individuals). The body composition measurement was conducted by the bioelectrical impedance method using BodyComp MF Plus and Bodygram Pro 3.0 software. Parameters assessed include body cell mass (BCM), total body water (TBW), extracellular body water (EBW), intracellular body water (IBW), fat mass (FM), fat-free mass (FFM), muscle mass (MM), and the body cell mass (BCM) index. A significant between-group difference was observed only for EBW, where subjects with ulcerative colitis were characterized by a higher mass of extracellular water (*p* = 0.0405). Individuals with ulcerative colitis were characterized by a lower BCM share than the control group (*p* = 0.0192). A comparative analysis of the body composition of male patients with ulcerative colitis with those who did not have ulcerative colitis revealed only minor differences. The risk of malnutrition, assessed using both BMI and bioelectrical impedance, in men with ulcerative colitis in remission is the same as in healthy males in the matched general population.

## 1. Introduction

The main symptoms of inflammatory bowel disease (IBD) include diarrhea (with severity dependent on the disease course), blood and mucus in stool, painful tenesmus, abdominal cramps, fever, fatigue, as well as loss of appetite and body mass [1]. Diarrhea occurs as a result of the colonic dysfunction with regard to water and mineral absorption as well as stool formation [2]. The abovementioned symptoms may influence the nutritional status and, consequently, they may affect the general well-being of patients.

Clinical methods that are commonly applied in other chronic diseases for the assessment of nutritional status are often useless in IBD [3]. Results of biochemical analysis, such as levels of albumin, prealbumin, or transferrin, in IBD individuals may be influenced not only by the nutritional status, but also by the chronic inflammatory process [4]. Furthermore, the skinfold measurement may not be useful because of the necessity to monitor slight changes of nutritional status, which are often below the detection threshold of the method [5].

The methods which are most adequate for nutritional status assessment in the majority of IBD individuals are simple indices, such as body mass loss, body mass index (BMI) [3], or Rohrer’s index [6]. Moreover, it is essential to simultaneously analyze the typical daily intake of nutrients for comparison with recommended values for a more precise analysis of the nutritional status [3]. However, to analyze subtle differences such as minor changes of nutritional status, an assessment based only on body mass and height—even when accompanied by dietetic assessment—may be insufficient. Therefore, body composition analysis using the bioelectrical impedance method is, in practice, a useful additional assessment despite the requirement for adequate preparation of patients and equipment [7,8,9].

Body composition is a gender-dependent factor [10]; however, it is dependent on age [11] and presence of chronic diseases as well [12]. Consequentially, a separate assessment for male and female patients, together with the use of gender- and age-dependent population-based predictive algorithms and reference values, is essential [13]. At present, however, IBD-dependent predictive algorithms and reference values are nonexistent. Therefore, a number of published research on body composition assessment in IBD individuals is very limited. Moreover, a significant share of the published research that used the bioelectrical impedance method have undertaken combined analysis of male and female patients [7,9] or those with Crohn’s disease and ulcerative colitis [14].

Taking these factors into account, it may be valuable to compare the nutritional status of IBD patients with healthy ones in gender-specific subgroups. For male patients, such a comparison may be especially interesting as male IBD patients rarely undertake any dietetic modifications or other non-pharmacological treatment options [15]. Therefore, their body composition is unrelated to a strict diet, but rather, to factors directly associated with the disease course.

The aim of the presented research was to analyze the nutritional status of male subjects with ulcerative colitis in remission by using body composition that was assessed by bioelectrical impedance measurement against a gender-matched control group.

## 2. Experimental Section

### 2.1. The Design of the Study

The study was conducted at the Department of Dietetics, Warsaw University of Life Sciences (WULS-SGGW). The body composition (assessed by bioelectrical impedance measurement) of remission ulcerative colitis male individuals and pair-matched control male individuals group were analyzed. The study was conducted according to the guidelines laid down in the Declaration of Helsinki and all procedures involving human subjects were approved by the Bioethical Commission of the Central Clinical Hospital of the Ministry of Interior in Warsaw (No 35/2009) and the Bioethical Commission of the National Food and Nutrition Institute (No 1604/2009). Written informed consent was provided by all participants.

### 2.2. Study Participants

The study included male patients with ulcerative colitis in remission who were recruited and monitored at the three Warsaw Gastroenterology Outpatient Clinics: Gastroenterology Outpatient Clinic, Maria Skłodowska-Curie Memorial Cancer Center and Institute of Oncology; the Gastroenterology Outpatient Clinic, Central Clinical Hospital of the Ministry of Interior and Administration in Warsaw; and Gastroenterology Outpatient Clinic, Public Central Teaching Hospital, Warsaw. Patients diagnosed with ulcerative colitis at these outpatient clinics were invited to participate in the study and screened on the basis of the inclusion and exclusion criteria. Forty-four male patients in remission with ulcerative colitis were recruited for this study; simultaneously, a matched control group of 44 male individuals without IBDs was recruited from four Warsaw general medical centers. Healthy volunteers were invited through study advertisements and later screened as per the inclusion and exclusion criteria.

Inclusion criteria for subjects with ulcerative colitis were: outpatients with endoscopically diagnosed ulcerative colitis and confirmed remission (clinical remission: no diarrhea and no blood in stool; endoscopic remission: image without any changes or disappearance of vascular network, erythema, inflammatory polyps allowed), assessed by the Mayo Scoring System and the Rachmilewitz Index for ulcerative colitis activity, as in previous own studies [16,17]; age 18–80 years; clinical remission for at least six weeks; and stable drug dose for at least six weeks.

All subjects with ulcerative colitis were on standard treatment. The mean duration of treatment by a gastroenterologist was 6 years (range 1–17 years, nonparametric distribution), and the median of number of hospitalizations was 0.25 per year (range 0–3 per year, nonparametric distribution).

The control group was matched (pair-matching method) by age and concurrent diseases that were not complications of ulcerative colitis (i.e., cardiovascular disorders, gastrointestinal diseases other than IBDs, osteoarthropathic diseases, diabetes, anemia, allergies). Wherever a disease was indicated as a complication of ulcerative colitis, it was excluded from the pair-matching procedure. During matching, the body mass and BMI were not taken into account as factors that may influence body composition; this has been an accepted practice in studies by other authors [18].

Exclusion criteria for the ulcerative colitis and control group were patients with: metal orthopedic prosthesis/implants and pacemakers; epilepsy; limb amputations; abnormal limb or trunk conditions (e.g., evident scoliosis); skin lesions on the right side of the body where the measurement was to be conducted.

### 2.3. Body Composition Measurement

To our knowledge this is the first study to compare the body composition measurements in a group of ulcerative colitis males and pair-matched group of healthy male individuals.

The body composition measurement was conducted by the bioelectrical impedance method using Body Comp MF Plus (Akern Srl, Pontassieve, Italy). According to the widely accepted methodology [19,20], participants were in a fasting state and asked to abstain from alcohol or physical exercise for 24 h prior to measurement. Immediately prior to the measurement, subjects were asked to void bladder, undress up to light underwear, and remove metallic accessories (e.g., watch or jewelry) to enable more precise measurement.

Before the measurement, both height (accuracy ± 0.5 cm) and weight (accuracy ± 0.5 kg) were measured using standard devices (stadiometer and a calibrated professional medical weighing scale). The values thus obtained were used to calculate the BMI using the Quetelet equation (body mass (kg)/height^2^ (m^2^)).

All measurements were taken from the right side of the body. The skin on the dorsal surface of the foot and hand was cleaned with rubbing alcohol and disposable electrodes, compliant with ISO 10993–1:2003 standards, were applied—two on the dorsal surface of the right foot and two on the dorsal surface of the right hand; the minimum distance between two electrodes was 5 cm.

Values were recorded after the subject had been supine for 5–10 min, with arms straightened and separated from the trunk by approximately 30° and the legs straightened and separated by approximately 45°. There was to be no contact of body with the metal frame of the bed.

The values of impedance at 5 kHz, 50 kHz, and 100 kHz (Ω) were recorded. From these, by using Bodycomp MF Plus 2.0 software (Akern Srl, Pontassieve, Italy), an estimation of the assessed parameters was conducted, while predictive equations were applied. Parameters assessed include body cell mass (BCM), total body water (TBW), extracellular body water (EBW), intracellular body water (IBW), fat mass (FM), fat-free mass (FFM), muscle mass (MM), and the body cell mass (BCM) index. For some of the parameters assessed, there are reference values dependent on race, gender, age, weight, and height; therefore, these factors were used for the assessment and interpretation of the results of BCM [21], TBW [22], EBW [23], FM [24], and FFM [24], and the 50th percentile or mean population values were taken as the standard reference. The BMI was assessed by the WHO criteria [25,26]: <18.5 kg/m^2^—malnutrition; <18.5; 25 kg/m^2^)—appropriate body mass; <25 to 30 kg/m^2^) —overweight; and ≥30 kg/m^2^—obesity. The BCM index was assessed according to the manufacturer’s instructions (Akern Srl, Pontassieve, Italy): <8—malnutrition; <8; 15)—typical body composition; ≥15—muscular body.

### 2.4. Statistical Analysis

The obtained data are presented as means ± standard deviations (SD) with minimum, maximum and median values. The distribution of the analyzed factors was verified, using the Shapiro-Wilk test. The difference of BMI and body composition parameters between groups were analyzed using Student’s *t*-test (for parametric distribution) or the Mann–Whitney *U* test (for nonparametric distribution). The difference of number of individuals in categories was compared between groups using chi^2^ test. The level of significance *p* ≤ 0.05 was accepted. The statistical analysis was carried out, using the Statistica software version 8.0 (StatSoft Inc., Tulsa, OK, USA) and Statgraphics Plus for Windows 4.0 (Statgraphics Technologies Inc., The Plains, VA, USA).

## 3. Results

The median age of the ulcerative colitis subgroup was 37 years (range 19–75 years, nonparametric distribution), which was matched for the control group. Since the control group was matched for concurrent diseases that were not complications of ulcerative colitis, their frequency was not compared between the groups. However, the frequency of all concurrent diseases (excluding ulcerative colitis, but including its complications) was compared. For majority of patients with ulcerative colitis that were analyzed, 1–2 concurrent diseases were observed; however, in the control group, no concurrent diseases were diagnosed in the majority of subjects (*p* = 0.0416, chi^2^ test). Compared to the control group, the group with ulcerative colitis had a higher incidence of gastrointestinal diseases (*p* = 0.0002, chi^2^ test) and anemia (*p* = 0.0007, chi^2^ test). Furthermore, a higher number of bowel movements (median 14 per week, range 7–42, nonparametric distribution) was reported in the group with ulcerative colitis in remission versus the control group (median seven per week, range 5–25, nonparametric distribution) (*p* = 0.0000, Mann–Whitney *U* test).

The general demographic characteristics was described, specifically educational background of the study group with ulcerative colitis in remission (7%—primary education, 36%—secondary education, and 57%—higher education), and it was found similar for the control group (*p* = 0.1394, chi^2^ test).

The results of body composition measured using the bioimpedance method for both study groups are presented in Table 1. A significant between-group difference was observed only for EBW, where subjects with ulcerative colitis were characterized by a higher mass of extracellular water (*p* = 0.0405). However, on recalculating the per body mass/TBW mass, the observed difference was not significant (Table 2). Similarly, on presenting the analyzed factors as relative results of body composition, significant differences in the BCM share were revealed and individuals with ulcerative colitis were characterized by a lower share than the control group (*p* = 0.0192).

Both BMI and relative results of body composition, in comparison with reference values, are presented in the Table 3; there were no significant between-group differences.

An additional analysis was conducted to compare the body composition of individuals with ulcerative colitis who had appropriate BMI (18.5–25 kg/m^2^) against BMI-matched subjects in the control group. Although a difference of TBW was observed (44.7 L and 41.9 L for the ulcerative colitis and control groups, respectively; *p* = 0.0111, Mann–Whitney *U* test), there were no differences in the EBW and IBW.

## 4. Discussion

Despite the scarcity of published literature on body composition assessment by using the bioelectrical impedance method in patients with ulcerative colitis, there are some interesting published data which allow a comparison of our study results with the observations of other authors. In the Italian research, the FFM and FM of 25 male and 14 female patients with ulcerative colitis were assessed and compared with results from patients with Crohn’s disease [8]. In the Swedish research, a study analyzed the TBW, EBW, IBW, and FFM for nine male and 12 female IBD patients, where the analysis was undertaken on a combined cohort of patients with ulcerative colitis and Crohn’s disease [14]. In Dutch research, the FFM and FM of 14 male and 18 female patients with ulcerative colitis were compared with those of the control group [27]. In recently published Polish research, the FFM and FM of 34 patients with ulcerative colitis and 25 patients with Crohn’s disease were assessed and compared with the values obtained from healthy subjects; however, the analyzed groups comprised only children and adolescents [28,29].

The control group was recruited without BMI matching; however, the BMI of the control group did not differ from that of the group with ulcerative colitis. Furthermore, the control group was recruited while taking into account the age and concurrent diseases that were not complications of ulcerative colitis; it was concluded that BMI and other factors were characterized by typical distribution for men. The lack of a significant difference of BMI in the two study groups may imply that, in this study population, ulcerative colitis did not cause a decrease in body mass; furthermore, this could be interpreted as being associated with either low intensity of diarrhea symptoms with ulcerative colitis or a lack of nutritional modifications because of ulcerative colitis. Another explanation for this may be that there was compensation of the excessive number of bowel movements by higher nutritional intake. In general, this could be confirmed by results of research by other authors, who conclude that men with ulcerative colitis rarely apply therapeutic options other than pharmacological treatment, including dietetic modifications [15].

The observed lack of difference in BMI between our study groups corresponds with the results of the study by Back et al. [30], who reported a high prevalence of overweight and obesity in patients with IBD. Similarly, in the study of DeClercq et al. [31], participants with ulcerative colitis were characterized by significantly higher body mass and BMI than the control group. However, such observations may be typical especially in ulcerative colitis and not in Crohn’s disease, because individuals with Crohn’s disease tend to have a lower lean mass than those with ulcerative colitis [32]. In addition, in the research of Capristo et al. [8], a lower FM was observed in the group with Crohn’s disease than in the group with ulcerative colitis. Moreover, it has been observed that, independently of the extent of disease in patients with ulcerative colitis, there is an inadequate intake of nutrients even if the energy value of the diet may be adequate [33]; therefore, this may be associated with nutrient deficits, but without lower BMI.

Research by other authors has shown a tendency to excessive body mass in patients with ulcerative colitis in remission, especially if the disease was diagnosed at least 10 years earlier and the patients had modified their diet according to their individually perceived nutritional limitations [34]. The average BMI in patients with ulcerative colitis in remission in other analyses was higher than 25 kg/m^2^ [35], and indicated malnutrition in less than 2% of individuals [36]. Furthermore, in other Polish research, similar observations were made; Poniewierka et al. [6] noted BMI higher than 25 kg/m^2^ for 34.5% of their patients with ulcerative colitis, while 8.6% had BMI higher than 30 kg/m^2^ that was confirmed by the Rohrer’s index; the corpulent body build was reported for 36% of the studied male subjects. Thus, the results of a comparison between patients with ulcerative colitis and Crohn’s disease in their study may indicate that, as it was previously suggested, only patients with Crohn’s disease, and not those with ulcerative colitis, are characterized by altered body composition [6].

The indicated excessive body mass in the analyzed male population with ulcerative colitis is associated with a significant proportion of subjects with excessive FM and low FFM share. Therefore, it may be concluded that fat accumulation mainly contributed to the observed excessive body mass. However, it should be mentioned that in other research on male patients with ulcerative colitis, the bioelectrical impedance method tended to underestimate the FFM as compared with the doubly labeled water reference method [14]; this could indicate an underestimation of the FFM concurrent with an overestimation of FM in the study population.

With regard to reference values, men in this study population with ulcerative colitis were characterized by excessive body mass, because the BMI of the majority was higher than the recommended range. Although the published literature report on malnutrition being a common observation in patients with ulcerative colitis [35], the BMI of the study population with ulcerative colitis do not concur with this conclusion. However, considering the fact that the analyzed cases were in remission, they may have had a higher BMI than expected. Furthermore, in the review article by Gerasimidis et al. [37], they reported recent data that suggested not only fewer patients than expected are malnourished, but also a large share of the population with ulcerative colitis is overweight or even obese.

In our study population, we observed higher BMI, FM, and FFM in subjects with ulcerative colitis in comparison with that reported in other research work [8,27]; this was attributable to the higher body mass in the analyzed patient group. When relative results of body composition (recalculated per body mass/TBW mass) were compared, similar results were observed for MM and FM in the analyzed group and the groups analyzed by other researchers, which may indicate typical results of body composition proportions [8].

Furthermore, a comparison of the IBW in this study with the results obtained in others with both male and female patients with ulcerative colitis indicated similar results [9]; however, no definite conclusions could be drawn when comparing our results with study populations that analyzed a combined population of male and female patients. However, in other research conducted in male patients with ulcerative colitis [14], similar findings as in our study were reported for TBW, EBW, and IBW. This could possibly confirm that the results obtained are typical for men with ulcerative colitis and that, in general, the results of body composition of men with ulcerative colitis are typical of the matched male population without ulcerative colitis.

The only minor differences that were observed between male patients with ulcerative colitis and the control group were associated with EBW and BCM. In fact, these compared values were not measured, but rather, predicted whereas the accuracy of measurements conducted using equations that are nonspecific for patients with ulcerative colitis or IBD is questionable. The lack of specific dedicated equations must be indicated as a limitation of this study. Moreover, a comparison of the body composition parameters with the reference values indicated a lack of significant differences, indicating the need to conduct further studies in larger groups of patients with ulcerative colitis in remission.

## 5. Conclusions

A comparative analysis of the body composition of male patients with ulcerative colitis with those who did not have ulcerative colitis revealed only minor differences—mainly, with regard to EBW and BCM. Therefore, male patients with ulcerative colitis in remission are characterized by similar body composition as the matched population without IBDs. Thus, the risk of malnutrition, assessed using both BMI and bioelectrical impedance, in men with ulcerative colitis in remission is the same as in healthy males in the matched general population.

## Figures and Tables

**Table 1:** The results of body composition measured using bioimpedance method for ulcerative colitis and control groups of male individuals.

Parameter	Ulcerative Colitis Group of Male Individuals				Control Group of Male Individuals				*p*-Value **
	Mean ± SD	Median	Min	Max	Mean ± SD	Median	Min	Max	
Z_5_ (Ω)	541.2 ± 60.5	544.0	426.0	665.0	556.3 ± 76.9	552.0	409.0	707.0	0.4424
Z_50_ (Ω)	476.9 ± 65.3	486.0	384.0	575.0	492.4 ± 75.4	495.5	398.0	619.0	0.3494
Z_100_ (Ω)	451.3 ± 65.6	448.0	363.0	546.0	468.3 ± 74.3	470.5	375.0	589.0	0.2602
BCM (kg)	30.4 ± 4.8	29.9	15.9	40.7	31.9 ± 7.3	33.5	13.3	45.7	0.2366
TBW (L)	46.2 ± 4.8	45.9	34.0	57.8	45.2 ± 5.5	44.3	35.3	58.7	0.3687
EBW (L)	20.6 ± 2.6	20.8	14.0	26.3	19.9 ± 4.1	19.1*	14.9	32.0	0.0405
IBW (L)	25.6 ± 3.6	25.4	17.2	33.9	25.3 ± 4.0	25.8	16.9	35.3	0.7555
FM (kg)	18.4 ± 7.1	16.8 *	8.5	33.0	17.5 ± 6.7	17.3	5.9	33.7	0.6887
FFM (kg)	62.3 ± 7.0	61.7	46.5	79.0	61.3 ± 7.6	61.2	44.8	80.2	0.5240
MM (kg)	37.6 ± 5.4	37.1	21.8	49.8	39.3 ± 8.1	41.8	19.7	55.4	0.2511
Height (cm)	178.0 ± 7.0	178.0	165.0	190.0	176.9 ± 6.2	176.0	165.0	192.0	0.5980
Weight (kg)	80.8 ± 11.22	78.5 *	63.0	112.0	78.8 ± 11.9	76.5	57.5	104.0	0.3900

SD—standard deviation; Z_5_—impedance at 5 kHz; Z_50_—impedance at 50 kHz; Z_100_—impedance at 100 kHz; BCM—body cell mass; TBW—total body water; EBW—extracellular body water; IBW—intracellular body water; FM—fat mass; FFM—fat-free mass; MM—muscle mass; * nonparametric distribution in a group; ** *p*-Value for the Student’s *t*-test/Mann–Whitney *U* test.

**Table 2:** The BMI and relative results of body composition measured using bioimpedance method for ulcerative colitis and control groups of male individuals.

Parameter	Ulcerative Colitis Group of Male Individuals				Control Group of Male Individuals				*p*-Value **
	Mean ± SD	Median	Min	Max	Mean ± SD	Median	Min	Max	
BMI (kg/m^2^)	25.6 ± 3.6	25.7	19.4	34.6	25.2 ± 3.4	24.5 *	20.0	32.9	0.5424
BCM (% of body mass)	48.9 ± 6.6	48.9	27.9	61.2	52.0 ± 10.6	53.4 *	20.0	64.5	0.0192
TBW (% of body mass)	57.0 ± 6.5	57.6 *	27.9	65.8	58.0 ± 4.3	57.1 *	50.4	67.4	0.9534
EBW (% of total body water)	44.6 ± 4.3	43.6 *	39.0	59.8	43.9 ± 6.4	41.9 *	36.2	63.2	0.0741
IBW (% of total body water)	55.4 ± 4.3	56.5 *	40.2	61.0	56.1 ± 6.4	58.2 *	36.8	63.8	0.0734
FM (% of body mass)	22.4 ± 6.5	21.4	12.2	35.4	21.7 ± 6.1	22.7	8.0	32.0	0.4859
FFM (% of body mass)	77.7 ± 6.5	78.6	64.6	87.8	78.3 ± 6.1	77.3 *	68.0	92.0	0.9767
MM (% of body mass)	47.7 ± 9.3	47.0 *	27.3	86.3	50.3 ± 9.6	51.4	21.0	68.8	0.0721
BCM index (-)	9.6 ± 1.5	9.7	5.5	13.1	10.2 ± 2.2	10.5	3.7	14.7	0.1446

SD—standard deviation; BMI—body mass index; BCM—body cell mass; TBW—total body water; EBW—extracellular body water; IBW—intracellular body water; FM—fat mass; FFM—fat-free mass; MM—muscle mass; BCM index—body cell mass index; * nonparametric distribution in a group; ** *p*-Value for the Student’s *t*-test/Mann–Whitney *U* test.

**Table 3:** BMI and relative results of body composition in comparison with reference values – number of individuals characterized by values in categories, for ulcerative colitis and control groups of male individuals.

Parameter	Ulcerative Colitis Group of Male Individuals (% of Individuals)			Control Group of Male Individuals (% of Individuals)			*p*-Value *
	Lower than Reference Value **	Reference Value **	Higher than Reference Value **	Lower than Reference Value **	Reference Value **	Higher than Reference Value **	
BCM (kg)	72.7	-	27.3	52.3	-	47.7	0.0781
TBW (L)	50.0	-	50.0	59.0	-	41.0	0.5207
EBW (L)	63.6	-	36.4	70.4	-	29.6	0.6501
FM (kg)	36.4	-	63.6	34.1	-	65.9	1.0000
FFM (kg)	34.1	-	65.9	38.6	-	61.4	0.8246
BMI (kg/m^2^)	0.0	43.2	56.8	0.0	52.3	47.7	0.5220
BCM index (-)	13.6	86.4	0.0	15.9	84.1	0.0	1.0000

BMI—body mass index; BCM—body cell mass; TBW—total body water; EBW—extracellular body water; IBW—intracellular body water; FM—fat mass; FFM—fat-free mass; MM—muscle mass, BMR—basal metabolic rate; BMC index—body cell mass index; * *p*-Value for chi^2^ test. ** reference values of BCM [21], TBW [22], EBW [23], FM [24] and FFM [24], for the 50th percentile or mean population values.
